# T2 relaxation time for intervertebral disc degeneration in patients with upper back pain: initial results on the clinical use of 3.0 Tesla MRI

**DOI:** 10.1186/s12880-017-0182-z

**Published:** 2017-01-31

**Authors:** Raoying Xie, linhui Ruan, Lei chen, Kai Zhou, Jiandong Yuan, Wei Ji, Guangjian Jing, Xiaojing Huang, Qinglei Shi, Chun Chen

**Affiliations:** 1 Department of Orthopaedics, the First Affiliated Hospital, Wenzhou Medical University, Nan baixiang Road, Shangcai Village, Wenzhou, 325000 Zhejiang People’s Republic of China; 2 Department of Radiation and chemotherapy division, the First Affiliated Hospital, Wenzhou Medical University, Wenzhou, Zhejiang People’s Republic of China; 3 Department of Neurosurgery, the First Affiliated Hospital of Wenzhou Medical University, Wenzhou, Zhejiang People’s Republic of China; 4 Department of Orthopaedics, Navy General Hospital, Beijing, People’s Republic of China; 5 Siemens Ltd, China Healthcare Sector MR Business Group, Beijing, People’s Republic of China

**Keywords:** Intervertebral Disc Degeneration, Cervical, T2 Relaxation Time, Magnetic Resonance Imaging, Cervicothoracic Junction

## Abstract

**Background:**

Magnetic resonance imaging (MRI) is a useful non-invasive tool for evaluating abnormalities of intervertebral discs. However, there are few studies which applied functional MRI techniques to investigate degenerative changes in cervical and cervicothoracic junction (CTJ) spine among adults. The aim of this study was to compare T2 relaxation time measurement evaluation with morphological grading for assessing cervical and CTJ intervertebral discs (IVD) in the patients suffering neck, shoulder, and upper back pain.

**Methods:**

Sixty-three patients (378 IVDs) and 60 asymptomatic volunteers (360 IVDs) of the cervical and CTJ discs were assessed using a 3.0 T magnetic resonance imaging (MRI) protocol, including an sagittal T2 relaxation time protocol. The relaxation time values of the nucleus pulposus (NP) were recorded and all discs were visually graded according to Pfirrman’s grading system. The correlation between T2 relaxation time values and qualitative clinical grading of degeneration, patient age, sex and anatomic level were analyzed

**Results:**

There is a clear trend of decreasing mean T2 values of the NP associate with increasing Pfirrmann grades (C2-T1) for both patients and asymptotic volunteers. Significant T2 differences were seen among grades I-V (*P* < 0.05). However, grade V was not observed in the CTJ. Linear correlation analysis revealed a strong negative association between T2 values of the NP and Pfirrmann grade (*r* = −0.588, *r* = −0.808) of C2-7 and C7T1. Age were also significantly correlated NP T2 values (*r* = −0.525, *r* = −0.723) for patients and volunteers. Moreover, the receiver operating characteristic analysis for average measures in a range from 0.70-0.79 (C2-7) to 0.84-0.89 (C7T1) for patients.

**Conclusions:**

T2 quantitation provides a more sensitive and robust approach for detecting and characterizing the early stage of IVD degeneration and age-associated disc changes.

**Electronic supplementary material:**

The online version of this article (doi:10.1186/s12880-017-0182-z) contains supplementary material, which is available to authorized users.

## Background

Cervical intervertebral disc (IVD) degeneration changes range from 5% to 10% in people between the ages of 20 and 30 years, to more than 50% by 45 years of age. Approximately, 90% of men over the age of 50 and 90% of women over the age of 60 have radiographic evidence of cervical spondylosis [1]. Although there are many factors that can cause chronic neck, shoulder, and upper back pain [2, 3], degeneration of the cervical spine is the most common condition contribution. Moreover, a degenerative IVD in the cervicothoracic junction (CTJ) can also result in above syndromes [4].

Magnetic resonance imaging (MRI) is a promising non-invasive examining tool for evaluating abnormalities of IVD. Age and degeneration can be reflected by signal variation of the discs on T2-weighted images (T2WIs), which allows for the determination of degree of disc degeneration [5]. Specifically, changes in MRI signal strength, which related to water and proteoglycan (PG) content in the nucleus pulposus (NP), can indicate disc degeneration according to Pfirrmann grading system [5, 6]. However, a reliable quantification characterized by loss of water or PG in an intact disc is not provided in such system to evaluate the degenerative degree in early intervertebral disc degeneration (IVDD) stages.

Recently, several quantitative MRI techniques for evaluation of IVD have been used for evaluating disc degeneration [7–9]. In particular, The T2 relaxation time is the decay constant for T2 signal intensity (SI) in MRI which is an intrinsic property of the tissue reflecting the molecular environment created in the disc by water, proteins, fat, collagen, and other solutes [8]. Increased T2 relaxation times is associated with increased disc water or glycosaminoglycan content, the primary component of PG [8, 9]. A good correlation of T2 values and water content in lumbar IVD tissue has been the subject of many previous studies [10–13] and has been widely validated. In cervical spine, our research firstly evaluated early cervical IVDD quantified by T2 values MRI in asymptomatic young adults [11]. The results in our study confirmed that T2 relaxation time can be potentially used as a clinical tool to identify early IVDD and to formalize a reliable quantitative scale. However, to our knowledge, there are few studies to investigate degenerative changes in cervical and CTJ spine of adults using this functional MRI technique.

Therefore, the purpose of this study was to use the quantitative T2 relaxation time measurement of 3.0 T MRI to evaluate the degree of cervical and CTJ degenerative IVDs, comparing with Pfirrmann grading in a larger cohort of young-to-middle-aged patients with neck, shoulder, and upper back pain.

## Methods

### Ethics statement and study sample

The study was approved by the institutional review board of the First Affiliated Hospital, Wenzhou Medical University and all participants provided written informed consent prior to enrollment. This study involved 63 patients (26 male, 37 female, average age, 39.88 ± 12.47 years; range: 20–76 Y/O., 378 discs in total) and 60 asymptomatic volunteers (32 male, 28 female; average age, 37.64 ± 10.65 years, range: 22–70 Y/O., 360 discs in total) in our initial study, who accepted MRI scan of their cervical spine from C2 to T1. They were consecutively recruited and evaluated from September 2014 to May 2015. The subjects were excluded if they had diabetes mellitus, major systemic disease, serious illness (e.g., tumor, infection), back surgery, spinal fractures, or osteoporosis. They consisted of patients with back pain and/or brachial plexus neuralgia. The following inclusion criteria was applied: the patients had significant neurological symptoms, which were defined as upper back pain for > 2 weeks and severe enough to require physician consultation or treatment, including neck or arm weakness, numbness, or tingling [14]. In parallel, inclusion criteria of asymptomatic volunteers referenced are reported by Pfirrmann et al. [5], which observed the process of normal aging in the lumbar discs, as follows: 1) no upper back pain within the last 5 years, 2) never absent from work due to upper back pain or numbness, and 3) no history of consulting a physician due to upper back pain, arm weakness, numbness or tingling [11].

### MR image acquisition

A GE Signa HDx 3.0 T MRI machine (GE Healthcare, Milwaukee, WI, USA) with a spine coil was used for MRI scanning. All MR images in this study were obtained in the afternoon to minimize the diurnal variation of T2 values in the IVDs [15]. Sagittal T1-weighted fast spin echo (FSE), and sagittal, transversal, and axial T2-WIs-FSE sequences were used for morphological MRI. T2-WIs (Time repetition (TR) 2560 ms, Time echo (TE) 102 ms, received band width [RBW] ± 31.25 kHz, field of view [FOV] 24 × 24 cm, matrix 320 × 224, slice thickness/gap 3 mm/0.5 mm, number of excitations [NEX] 4, total scan time 2 min and 18 s) were obtained and a radiologist blindly ranked the severity of disc degeneration as grade I to V in the midsagittal section according to the Pfirrmann classification (see Additional file 1: Table S1). Thereafter, a T2 map was generated on the midsagittal section, which was chosen from sagittal sections of cervical midline, by using the optimal T2 values obtained at a sequence of 8 echo multi-spin echo (TR/first echo TE, last echo TE, 3000/8.5-67.9, RBW ±31.25 kHz, FOV 18 × 18 cm, matrix 256 × 160, slice thickness/gap 3 mm/0.5 mm, NEX 1, total scan time 4 min and 27 s). All T2 map images were generated on the Advantage Workstation (version 4.3, Functool; GE Healthcare, Milwaukee, WA, USA) [10]. However, the first echo from the multi-spin system was excluded to minimize the effect of the stimulated echo.

### Image analysis

**Fig. 1:**
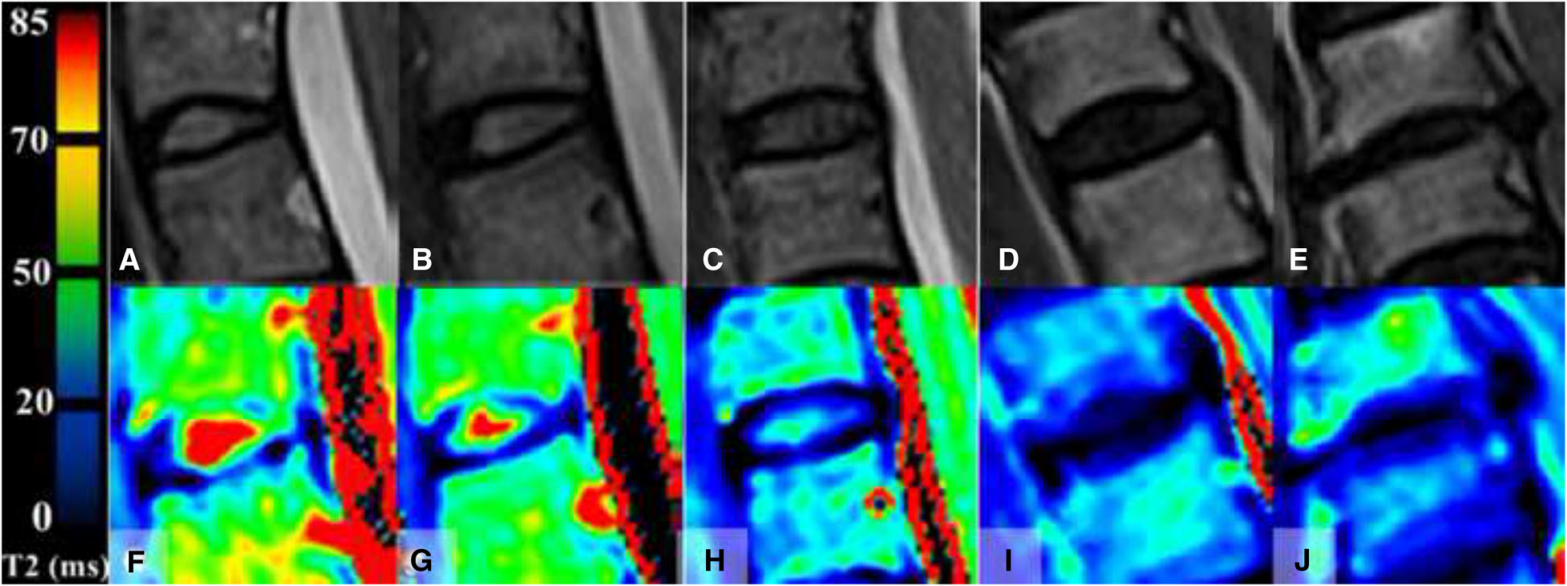
Sagittal T2-weighted FSE images and corresponding T2 maps of discs with different Pfirrmann grades. **a** and **f** grade I; (**b** and **g**) grade II; P (**c** and **h**) grade III; (**d** and **i**) grade IV; (**e** and **j**) grade V

Evaluation of all morphological images were performed by two radiologists in consensus, one with more than 10 years of experience and a special interest in musculoskeletal radiology, and the other one with more than 20 years of experience in orthopedic radiology. Though challenging to appraise cervical or lumbar MRI [13], no obvious transitional vertebrae were detected. For measurement, to evaluate regions of interest (ROIs) in a standardized and reproducible way, we decided to adopt a previous reported method [7, 16, 17]. By using T2-WI as reference, regions of interest (ROIs) were manually drawn over the T2 map of the discs by an orthopedic consultant (GJ) with 20 years’ experience reading MRI images. The geometrical center of the ellipses were defined to the intervertebral area ROIs only included the NP area. All ROIs were selected on the morphological images and transferred via “copy and paste” into the T2 maps with areas of 9.25 ± 1.38 mm^2^ to cover the NP using the software. All of data was measured by three times in order to reduce error. The exemplary Pfirrmann grades were shown in Fig. 1.

### Inter- and intraobserver analysis

The intra- and interobserver variability of the ROI evaluation was assessed. Two central sagittal slices of T2 map were evaluated as already described. The evaluation was performed by two independent observers with different skill levels (musculoskeletal radiologist with 15 years of experience [observer 1], orthopedic surgeon with 20 years of experience [observer 2]). In addition, observer A and B performed the same analysis twice, with an interval of one month, to test intraobserver agreement.

### Statistical analysis

Statistical analysis and graph drawing were performed using SPSS 19.0 (SPSS Inc., Chicago, IL, USA). Descriptive statistical data are given as mean ± standard deviation (Mean ± SD). To evaluate the reliability of Pfirrmann grading, intraobserver and interobserver agreements were graded through Kappa statistics. The degree of internal consistency was considered as: strong consistency, *k* ≥ 0.7; moderate consistency, 0.7 ≥ *k* > 0.4; and weak consistency *k* ≤ 0.4. One-way ANOVA and Student Newman-Keuls test were used to detect the statistical differences of T2 values between different Pfirrmann grades. Additionally, Receiver operating characteristic (ROC) curves were plotted to test the sensitivity and specificity of T2 measures in assessment of Pfirrmann grade. Areas under the ROC curves were calculated. The statistical difference of T2 measures used to evaluate Pfirrmann grade was determined by *U*-test. All tests above were considered significant with *P* < 0.05.

## Results

### Morphological MRI findings

Tables 1 and 2 show the characteristics of all analyzed discs sorted by the Pfirrmann degeneration grade of C2-T1 of patients and asymptomatic volunteers, respectively. According to the Pfirrmann grades, 13 and 48 discs (3.43%, 13.33%) were classified as grade I, 117 and 84 discs (30.95%, 23.33%) as grade II, 142 and 107 discs (37.56%, 29.72%) as grade III, 72 and 69 disc (19.04%, 19.16%) as grade IV, and 34 and 52 discs as grade V (8.99%, 14.44%).

**Table 1:** Degeneration grade according to Pfirrmann scale, and disc level of the cervical intervertebral discs of patients analyzed in study

Degeneration	Disc Levels						
Grade/N	C2-3	C3-4	C4-5	C5-6	C6-7	C7T1	Total
I	3	1	2	0	0	7	13
II	19	23	23	11	15	26	117
III	25	24	26	22	25	20	142
IV	10	9	11	18	14	10	72
V	6	6	1	12	9	0	34
Total	63	63	63	63	63	63	378

**Table 2:** T2 values in different level groups of asymptomatic volunteers

Pfirrmann	No.	T2 values (mean, ms)	No.	T2 values (mean, ms)
Grade		C2-7		C7T1
I	35	71.98 ± 12.34	13	85.45 ± 19.53
II	68	64.25 ± 11.28@	16	73.49 ± 15.44$
III	87	62.76 ± 9.37@	20	67.72 ± 9.71$%
IV	58	50.39 ± 10.75@#^	11	54.60 ± 8.11$%~
V	52	41.23 ± 7.73@#^&	—	—
Total/average	300	59.65 ± 9.55	60	68.71 ± 13.14

Mean values (±standard deviation) of T2 relaxation times in milliseconds in different compartments of the cervical intervertebral disc for different Pfirrmann groups are shown. NP, nucleus pulposus. @ Compared with T2 values of NP of grade I for C2-7, *P* < 0.000; #Compared with T2 values of NP of grade II for C2-7, *P* < 0.000; ^Compared with T2 values of NP of grade III for C2-7, *P* < 0.002; &Compared with T2 values of NP of grade V for C2-7, *P* < 0.05; $ Compared with T2 values of NP of grade I for C7T1, *P* < 0.004; %Compared with T2 values of NP of grade II for C7T1, *P* < 0.000; ~Compared with T2 values of NP of grade III for C7T1, *P* < 0.05

### Inter- and intraobserver agreement

Regarding the Pfirrmann grading reproducibility by the 2 readers, the intraobserver test yielded *k* values ranging from 0.826 (*p* = 0.000) to 0.793 (*P* = 0.000), whereas the interobserver test produced *k* values of 0.757 (*P* = 0.000).

### Results regarding pfirrmann classification

The mean value of T2 relaxation time at different regions of each Pfirrmann grade are presented in table 2 and table 3. As to patients, in the NP of C2-T1, T2 values tended to decrease with increasing Pfirrmann grades, and T2 values were significantly different when comparing grades I to V (*P* < 0.01, Table 3). However, Pfirrmann grade V was not observed among C7T1 discs. Regarding the T2 values of NP, significance was detected between T2 values of asymptomatic volunteers in the different Pfirrmann grades (Table 2). The Spearman correlation analysis indicated a strong negative correlation between the Pfirrmann grade and the T2 values of NP (*r* = −0.588; *P* = 0.000; *r* = −0.808, *P* = 0.000) of C2-7 and C7T1. Furthermore, significant correlation between age and T2 value of NP was demonstrated both in patient group (*r* = −0.525, *P* = 0.000) and volunteer group (*r* = −0.723, *P* = 0.000) . The overall mean value of T2 relaxation time of volunteer group was 59.65 ± 9.55 ms, which was significantly larger than its counterpart of patient group (46.19 ± 9.37 ms, 23.57% reduction, *P* < 0.01). Similarly, the mean value of T2 value of the 315 discs from patient group was significantly lower than that from volunteer group (57.02 ± 16.01 ms versus 68.71 ± 13.14 ms, 8.29% reduction, *P* < 0.05). At the same degeneration grade, the T2 values of patients were relatively smaller comparing to the T2 values of volunteers. As shown in table 4, the significant differences of sensitivity, specificity, area under the ROC curve (AUC) and T2 cut-off value between different Pfirrmann grades of C2-7 and C7T1, were demonstrated respectively (*P* < 0.05) by conducting *U*-test.

**Table 3:** T2 values for discs with different Pfirrmann grades

Grade	T2 (mean ± SD, ms, C2-7)		T2 (mean ± SD, ms, C7T1	
	No.	NP	No.	NP
I	6	62.99 ± 13.47	7	83.20 ± 17.59
II	91	50.75 ± 7.75@	26	58.46 ± 8.42$
III	122	44.83 ± 6.95@#	20	46.91 ± 4.98$%
IV	62	40.20 ± 5.62@#^	10	39.13 ± 5.13$~
V	34	34.93 ± 9.53@#^&		—
Total	315	46.19 ± 9.37	63	57.02 ± 16.01

Mean values (±standard deviation) of T2 relaxation times in milliseconds in different compartments of the cervical intervertebral disc for different Pfirrmann groups are shown. NP, nucleus pulposus. @ Compared with T2 values of NP of grade I for C2-7, *P* < 0.000; #Compared with T2 values of NP of grade II for C2-7, *P* < 0.000; ^Compared with T2 values of NP of grade III for C2-7, *P* < 0.002; &Compared with T2 values of NP of grade V for C2-7, *P* < 0.05; $ Compared with T2 values of NP of grade I for C7T1, *P* < 0.004; %Compared with T2 values of NP of grade II for C7T1, *P* < 0.000; ~Compared with T2 values of NP of grade III for C7T1, *P* < 0.05

## Discussion

In this prospective study, we developed and investigated an MRI method, applicable for conventional 3.0 Tesla units, for sagittal T2 mapping of cervical and CTJ IVDs in symptomatic patients and asymptomatic volunteers and then compared against the Pfirrmann grades which can be used in a clinical set-up. The results of this study suggest that negative correlations between T2 values and disc degeneration grades. More important, the results indicate that the IVDD of CTJ exist, range from grade I to IV. To the best of our knowledge, the present study is the first who use asymptomatic volunteers and symptomatic population to investigate the correlation between T2 values and Pfirrmann changes in the inner portion of cervical IVDs. This study suggests that T2 relaxation time can be sensitive to early degenerative changes in cervical IVDD.

MRI is commonly used in the clinical setting for examination of the degeneration of bone, cartilage and other tissues [6, 8, 13, 14]. Additionally, T2 mapping MRI has greater advantages over other methods of detecting matrix content [18]. The T2 relaxation time is able to provide superior information of early molecular and physiological alterations in IVDs as follows: 1) improving identification of IVDD in its early stages; 2) evaluating outcomes of biological treatments and 3) implementing continuous quantitative assessments of IVDD [8, 9, 12]. With the development of IVD tissue engineering, the T2 values can be used to follow regenerative progress of the NP, non-invasively.

Most previous studies emphasized T2 changes in the lumbar IVDD without consideration of cervical and CTJ [4]. Only our previous study reported a decrease of NP T2 values in asymptomatic healthy young adults [10]. Comparing with healthy young adults, the different grades of T2 values were smaller (grade I: 62.99 vs. 72.25 ms; II: 50.75 vs.59.36 ms; III: 44.83 vs. 51.73 ms) [10]. However, the T2 values of asymptomatic volunteers were relatively lager comparing with results in current study. The 3.0 T field used in the study and the different composition of subjects might contribute to the difference. The increase of regional T2 values will occur due to the magic angle effect when collagen fibers are oriented 54.7° relative to the orientation of the static magnetic field (B0) [19]. This effect can be shown inevitably in the annulus fibrosus, especially near its surface [20]. In view of this situation, the ROI in the present study only encompassed NP and inner annulus fibrosus. In our study, the T2 values of the discs with Pfirrmann grade IV-V (40.20 ms; 34.93 ms) were substantially lower than the discs with Pfirrmann grade I-III. There was a significant difference between the T2 values of the NPs with Pfirrmann grade IV and V. Previous studies have demonstrated that T2 values in the NP and AF decrease in accordance with lumbar IVDD, and, ultimately, difference in T2 SI between this two anatomical structures close to zero [21], which is consistent with results of the former study. Thus, comparison of T2 values of NP may also be valuable in the evaluation of disc degeneration. Moreover, the T2 values in cervical discs were lower than those previously results in healthy lumbar discs [12, 13, 22].

A degeneration IVD can also occur in CTJ. Strictly speaking, CTJ should contain the C7 vertebra, the disc between C7 and T1, and attachment of ligaments [4]. C7T1 disc herniation is responsible for less than 5% of all the cervical disc herniations [23]. In most of these patients, the common presenting symptom involving hand weakness and pain radiating to the lateral aspect of the hand were the same symptom as C2-7 IVD herniation [23]. Moreover, previous study reported that the incidence of the disc degeneration at C7T1 is 42% [24]. Another study reported cases that C7T1 IVDD occurred after C5-7 fusion [25]. Therefore, the clinical importance of the CTJ region may be related to the claimed concerning of region with neck and shoulder pain [26] and should be emphasized. In our study, T2 values of NP of C7T1 were higher than C2-7 with regard to Pfirrmann I and II, however, we found that most observations of C7T1 show no difference with the results of C2-7, The same as C2-7, the decreasing T2 values among different grades might attribute to decreasing water content and structural disorganization with severity increasing of disc degeneration.

Some previous studies showed no significant difference in T2 values between grade IV and V [12, 13] of lumbar which was in disagree with our present study. This difference may attribute to the various biochemical properties between cervical and lumbar IVDs, and measurement method. In accordance with our previous study, the T2 values were lower than former reported in lumbar discs [9, 13] which was a result of different biochemical changes among cervical, thoracic and lumbar [21].

It is important to realize that the correlation coefficients of T2 values of NP referring to grades and T2 values obtained in this study range between moderate and strong. This might be attributed to the relative large sample size. Moreover, the results also explained that the T2 value in the IVD is known to be sensitive to the composition of the collagen network structure and water content. Previous study reported that the T2 values are influenced by both rotational and translational motion which result in dipole-dipole interaction of water molecules in the collagen matrix [6].

Sekhon et al. confirmed a good to excellent correlation of T2 values, water content and PG content in IVD tissue [27]. Moreover, the inverse correlation has been observed between age and T2 values in the current study and several former studies [28]. As we known, it is difficult to distinguish a painful degenerative disc from one with age-related physiologic changes, therefore, we recruited a well-defined asymptomatic population for the current study for inspecting the effect of aging on T2 values. Given that previous studies recruited and analyzed IVDs of asymptomatic volunteers [11, 28], we had a much wider range of age resemble those researches and were a discrepancy compared to that in a previous study [29], which confirmed the inverse correlation between age and T2 values only for grade II discs in 20 patients with lumbar back pain or radiculopathy. However, it is important to point out that we did not consider the equivalence of age range and the balanced age distribution, that could introduce influence when analyze difference of disc degeneration for both the asymptomatic and symptomatic groups. Nevertheless, some authors also confirmed that T2 value is useful to evaluate IVDD after either distinguishing age groups or not [12].

The T2 maps can be generated and measured immediately after MRI scan, and the ROI analysis takes about 3 min for each patient. Moreover, an automated segmentation of the IVDs may facilitate acquiring promising initial data attribute to ongoing efforts in the future. Therefore, quantitative information reflecting the biochemical composition of IVDs can be provided without extensive increase in time.

ROC between each grade of the NP generated cut-off values obtaining from area under the curve (AUC) values for disc degeneration quantification via T2 SI. In this study, it was in consistent with previous study [12, 13] that all AUC values were within the moderate accuracy range, indicating a moderate level of reliability. These data demonstrated that this T2 value-based grade scale for the NP is a useful distinguishing system. Furthermore, the high correlation in the inter- and intraobserver analysis suggest that our standardized ROI positioning is adequately reproducible.

Naturally, this study has some limitations. Firstly, the major limitation of our study is no histological or biochemical analysis of IVDs performing. Secondly, partial volume effects still existed attributing to subjectivity and bias of ROIs selection. Thirdly, we did not compare some other imaging techniques such as T1p, T2*(star) or ADC and so on to results of T2 relaxation time [22, 30, 31], however, many studies had verified the practicability of this technique alone to diagnose lumbar IVDD [15, 32]. Additionally, the CTJ structure is not strictly defined. Some investigators involve thoracic 2 and sometimes thoracic 3 vertebrae and thoracic 2–3 disc and associated ligaments when discussing the CTJ, however, lesions involving the thoracic 2 and thoracic 3 vertebrae often face similar difficulties in reaching them through an anterior approach [4]. What’s more, a selection bias surely exists in the subject enrollment of all clinical prospective studies and cannot be fully removed. Exclusion criteria used in our study included diseases such as diabetes mellitus, major systemic disease, tumor, infection, back surgery, and so on, of which back pain is the main complain, and the IVDs of the patients who suffering those diseases are prone to degeneration [33, 34]. We established the same exclusion criteria for all participants in order to minimize the influence of selection bias. Furthermore, thoracic 2–3 IVDs should be studied in the future for better understanding of CTJ.

## Conclusions

In summary, our results proved a negative correlation between T2 values of cervical and CTJ IVDs and the Pfirrmann grades regarding IVDD. What should be highlighted is that we proposed the distinct cut-off values of the classification based on quantitative evaluation. Thus, the T2 value-based measurements of IVD water content and PG may be useful for estimating cervical and CTJ degenerative disc diseases.

## Additional file

Additional file 1: Table S1. Classification of intervertebral disc degeneration as reported by Pfirrmann et al. [6]. The table descripted the details of classification of intervertebral disc degeneration by Pfirrmann grades. (DOCX 16 kb)

## Abbreviations

AF: Annulus fibrosus
AUC: Area under the curve
C: Cervical
CTJ: Cervicothoracic junction
FOV: Field of view
FSE: Fast spin echo
IVD: Intervertebral disc
IVDD: Intervertebral disc degeneration
MRI: Magnetic resonance imaging
NEX: Number of excitations
NP: Nucleus pulposus
PG: Proteoglycan
RBW: Received band width
ROC: Receiver operating characteristic
ROI: Regions of interest
SD: Standard deviation.
SI: Signal intensity
T2WIs: T2-weighted images
TE: Echo time
TR: Repetition time

## References

[1] Grob D (1998). Surgery in the degenerative cervical spine. Spine.

[2] Trinh K, Cui X, Wang YJ (2010). Chinese herbal medicine for chronic neck pain due to cervical degenerative disc disease. Spine (Phila Pa 1976).

[3] Ehrmann Feldman D, Shrier I, Rossignol M, Abenhaim L (2002). Risk factors for the development of neck and upper limb pain in adolescents. Spine (Phila Pa 1976).

[4] Williams FM, Sambrook PN (2011). Neck and back pain and intervertebral disc degeneration: role of occupational factors. Best Pract Res Clin Rheumatol.

[5] Wang VY, Chou D (2007). The cervicothoracic junction. Neurosurg Clin N Am.

[6] Pfirrmann CW, Metzdorf A, Zanetti M, Hodler J, Boos N (2001). Magnetic resonance classification of lumbar intervertebral disc degeneration. Spine (Phila Pa 1976).

[7] Zou J, Yang H, Miyazaki M, Morishita Y, Wei F, McGovern S (2009). Dynamic bulging of intervertebral discs in the degenerative lumbar spine. Spine (Phila Pa 1976).

[8] Chen C, Jia Z, Han Z, Gu T, Li W, Li H (2015). Quantitative T2 relaxation time and magnetic transfer ratio predict endplate biochemical content of intervertebral disc degeneration in a canine model. BMC Musculoskelet Disord.

[9] Fields AJ, Han M, Krug R, Lotz JC (2015). Cartilaginous end plates: quantitative MR imaging with very short echo times-orientation dependence and correlation with biochemical composition. Radiology.

[10] Ogon I, Takebayashi T, Takashima H, Tanimoto K, Ida K, Yoshimoto M (2015). Analysis of chronic low back pain with magnetic resonance imaging T2 mapping of lumbar intervertebral disc. J Orthop Sci.

[11] Chen C, Huang M, Han Z, Shao L, Xie Y, Wu J (2014). Quantitative T2 magnetic resonance imaging compared to morphological grading of the early cervical intervertebral disc degeneration: an evaluation approach in asymptomatic young adults. PLoS One.

[12] Niu G, Yu X, Yang J, Wang R, Zhang S, Guo Y (2011). Apparent diffusion coefficient in normal and abnormal pattern of intervertebral lumbar discs: initial experience. J Biomed Res.

[13] Stelzeneder D, Welsch GH, Kovacs BK, Goed S, Paternostro-Sluga T, Vlychou M (2012). Quantitative T2 evaluation at 3.0T compared to morphological grading of the lumbar intervertebral disc: a standardized evaluation approach in patients with low back pain. Eur J Radiol.

[14] Takashima H, Takebayashi T, Yoshimoto M, Terashima Y, Tsuda H, Ida K (2012). Correlation between T2 relaxation time and intervertebral disk degeneration. Skeletal Radiol.

[15] Hanvold TN, Veiersted KB, Waersted M (2010). A prospective study of neck, shoulder, and upper back pain among technical school students entering working life. J Adolesc Health.

[16] Souza RB, Baum T, Wu S, Feeley BT, Kadel N, Li X (2012). Effects of unloading on knee articular cartilage T1rho and T2 magnetic resonance imaging relaxation times: a case series. J Orthop Sports Phys Ther.

[17] Nagashima M, Abe H, Amaya K, Matsumoto H, Yanaihara H, Nishiwaki Y (2012). A method for quantifying intervertebral disc signal intensity on T2-weighted imaging. Acta Radiol.

[18] Cai F, Wu XT, Xie XH, Wang F, Hong X, Zhuang SY (2015). Evaluation of intervertebral disc regeneration with implantation of bone marrow mesenchymal stem cells (BMSCs) using quantitative T2 mapping: a study in rabbits. Int Orthop.

[19] Wang YX, Zhao F, Griffith JF, Mok GS, Leung JC, Ahuja AT (2013). T1rho and T2 relaxation times for lumbar disc degeneration: an in vivo comparative study at 3.0-tesla MRI. Eur Radiol.

[20] Xia Y (2000). Magic-angle effect in magnetic resonance imaging of articular cartilage: a review. Invest Radiol.

[21] Van Breuseghem I, Bosmans HT, Elst LV, Maes F, Pans SD, Brys PP (2004). T2 mapping of human femorotibial cartilage with turbo mixed MR imaging at 1.5 T: feasibility. Radiology.

[22] Blumenkrantz G, Zuo J, Li X, Kornak J, Link TM, Majumdar S (2010). In vivo 3.0-tesla magnetic resonance T1rho and T2 relaxation mapping in subjects with intervertebral disc degeneration and clinical symptoms. Magn Reson Med.

[23] Chiu EJ, Newitt DC, Segal MR, Hu SS, Lotz JC, Majumdar S (2001). Magnetic resonance imaging measurement of relaxation and water diffusion in the human lumbar intervertebral disc under compression in vitro. Spine (Phila Pa 1976).

[24] An HS, Anderson PA, Haughton VM, Iatridis JC, Kang JD, Lotz JC (2004). Introduction: disc degeneration: summary. Spine (Phila Pa 1976).

[25] Maquer G, Laurent M, Brandejsky V, Pretterklieber ML, Zysset PK (2014). Finite element-based Non-linear normalization of human lumbar intervertebral disc stiffness to account for its morphology. J Biomech Eng.

[26] Post NH, Cooper PR, Frempong-Boadu AK, Costa ME (2006). Unique features of herniated discs at the cervicothoracic junction: clinical presentation, imaging, operative management, and outcome after anterior decompressive operation in 10 patients. Neurosurgery.

[27] Sekhon L (2005). Cervicothoracic junction arthroplasty after previous fusion surgery for adjacent segment degeneration: case report. Neurosurgery.

[28] Norlander S, Gustavsson BA, Lindell J, Nordgren B (1997). Reduced mobility in the cervico-thoracic motion segment--a risk factor for musculoskeletal neck-shoulder pain: a two-year prospective follow-up study. Scand J Rehabil Med.

[29] Wang YX, Griffith JF, Leung JC, Yuan J (2014). Age related reduction of T1rho and T2 magnetic resonance relaxation times of lumbar intervertebral disc. Quant. Imaging Med. Surg..

[30] Noriega DC, Marcia S, Ardura F, Lite IS, Marras M, Saba L (2016). Diffusion-weighted MRI assessment of adjacent disc degeneration after thoracolumbar vertebral fractures. Cardiovasc Intervent Radiol.

[31] Huang M, Guo Y, Ye Q, Chen L, Zhou K, Wang Q (2016). Correlation between T2* (T2 star) relaxation time and cervical intervertebral disc degeneration: an observational study. Medicine (Baltimore).

[32] Zobel BB, Vadala G, Del Vescovo R, Battisti S, Martina FM, Stellato L (2012). T1rho magnetic resonance imaging quantification of early lumbar intervertebral disc degeneration in healthy young adults. Spine (Phila Pa 1976).

[33] Lotan R, Oron A, Anekstein Y, Shalmon E, Mirovsky Y (2008). Lumbar stenosis and systemic diseases: is there any relevance?. J Spinal Disord Tech.

[34] Sierra-Jimenez G, Sanchez-Ortiz A, Aceves-Avila FJ, Hernandez-Rios G, Durán-Barragán S, Ramos-Remus C (2008). Tendinous and ligamentous derangements in systemic lupus erythematosus. J Rheumatol.

