# Supplementary material for: T2 relaxation time for intervertebral disc degeneration in patients with upper back pain: initial results on the clinical use of 3.0 Tesla MRI
Source: BMC Med Imaging. 2017 Jan 31;17:9. doi: 10.1186/s12880-017-0182-z (PMC5282844; doi:10.1186/s12880-017-0182-z)
Supplement: Additional file 1: Table S1. — Classification of intervertebral disc degeneration as reported by Pfirrmann et al. [6]. The table descripted the details of classification of intervertebral disc degeneration by Pfirrmann grades. (DOCX 16 kb) [file 12880_2017_182_MOESM1_ESM.docx]

Table S1. Classification of intervertebral disc degeneration as reported by Pfirrmann et al [6].

| Grade | Structure | Distinction of the  NP and AF | Signal intensity | Height of the IVD |
| --- | --- | --- | --- | --- |
| I | Homogeneous, bright white | Clear | Hyperintense, isointense to CSF | Normal |
| II | Heterogeneous with or without horizontal bands | Clear | Hyperintense, isointense to CSF | Normal |
| III | Heterogeneous, gray | Unclear | Intermediate | Normal to slightly decreased |
| IV | Heterogeneous, gray or black | Lost | Intermediate or hypointense | Normal to moderately decreased |
| V | Heterogeneous, black | Lost | Hypointense | Collapsed disc space |

CSF: cerebrospinal fluid; NP: nucleus pulposus; AF: annulus fibrosus; IVD: intervertebral disc.
